# Getting to zero HIV deaths: progress, challenges and ways forward

**DOI:** 10.7448/IAS.16.1.18927

**Published:** 2013-12-01

**Authors:** Nathan Ford, Marco Vitoria, Gottfried Hirnschall, Meg Doherty

**Affiliations:** HIV/AIDS Department, World Health Organization, Geneva, Switzerland

## Introduction

With the progressive increase in the global access to antiretroviral therapy (ART), there has been a major decline in HIV-related deaths over the past two decades. Before ART became widely available, people presenting at clinics with AIDS-defining illnesses would, on average, die within less than 1 year [1]. Today, access to ART is widespread, with some 9.7 million people estimated to be receiving ART globally by the end of 2012 [2]. The scale up of ART has averted an estimated 4.2 million deaths in low- and middle-income countries between 2002 and 2012 [2] and studies from high-income and low-income settings have concluded that, with timely access to ART, people living with HIV can expect a near-normal life expectancy [3–6].

**Figure 1:**
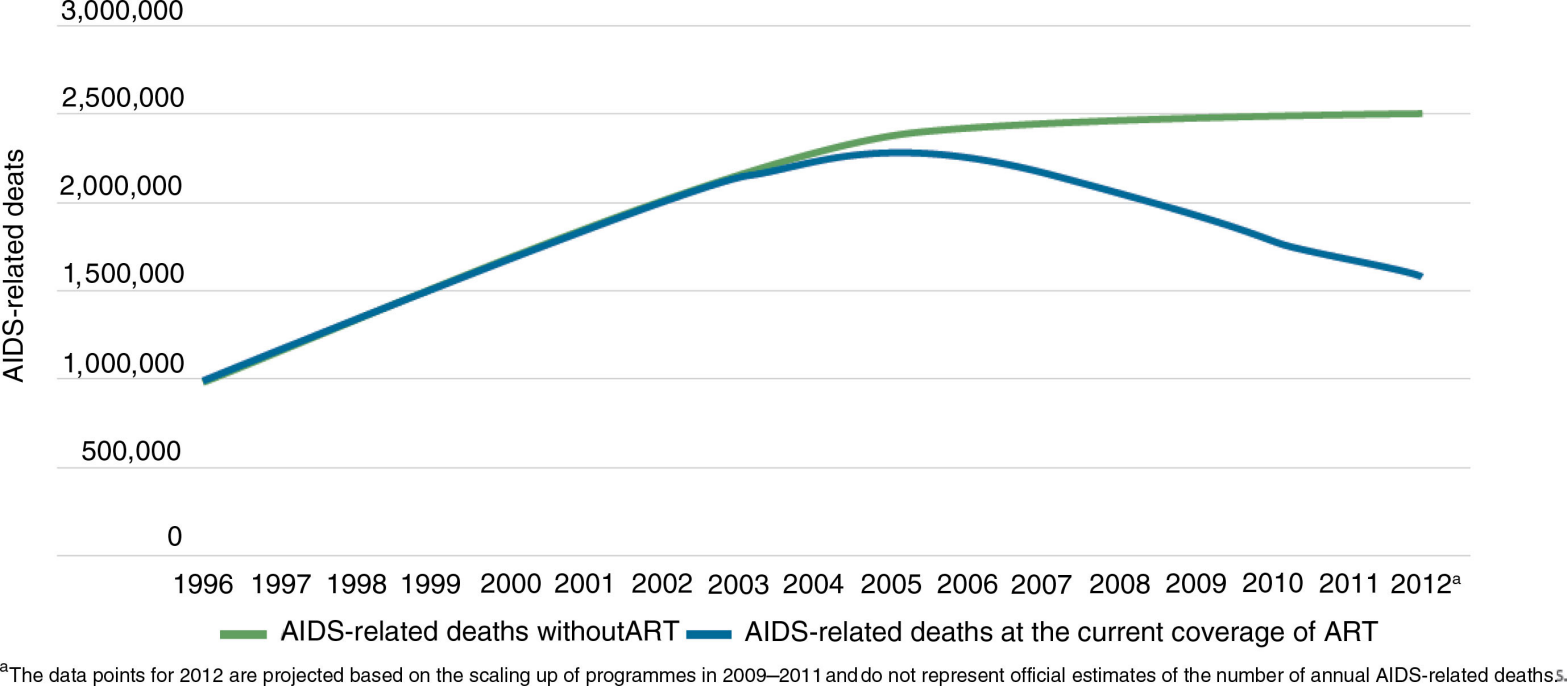
Annual number of people dying from HIV-related causes in low- and middle-income countries globally compared with a scenario of no antiretroviral therapy, 1996–2012.

Nevertheless, in 2012, an estimated 1.6 million people died of HIV-related causes [2], and HIV/AIDS still ranks in the top five global causes of disability-adjusted life years [7]. Thus, despite an important overall decline in HIV-related deaths over the past decade (Figure 1), much still needs to be done to get closer to the global target of zero deaths for HIV. There are four key challenges that must be addressed.

## The challenges

The first challenge is to continue to improve access to ART, with priority for those in urgent clinical need (CD4 cell count ≤350 cells/mm^3^ or the presence of severe HIV disease). Behind the impressive global trend of a linear increase in ART access, countries are making variable progress, with renewed efforts needed in some countries where treatment access appears to be stalling. Access to ART is also consistently lower for certain population groups, most notably children, men, adolescents and key affected populations (men who have sex with men, sex workers, people who inject drugs, transgender persons and migrants) [2].

A second and related challenge is that even among those who start ART, a high proportion continue to start late in their disease progression. Despite progressive improvements in HIV diagnosis and access to treatment and care, substantial numbers of patients continue to present for care only when they have reached an advanced stage of disease. A meta-analysis of data from 44 studies conducted in high-income countries reported that a mean annual CD4 increase of only 1.5 cells/mm^3^ was achieved at treatment presentation between 1992 and 2011 [8]. In high-income settings, any patient who presents with a CD4 cell count of ≤350 cells/mm^3^ is considered to be a “late presenter” [9]; studies from Europe report that around 50% of patients present late for care, with consequent increased risk of illness and death [10–12]. In resource-limited settings, patients present far later for care, on average with a CD4 cell count of <200 cells/mm^3^ or with an AIDS-defining event [2,13]; 20–40% start ART with a CD4 of <100 cells/mm^3^ [2]. Such late presentation is associated with lower survival [14], early mortality particularly associated with tuberculosis, and invasive bacterial and fungal infections [15,16].

Third, once on ART, efforts are needed to ensure that treatment remains effective. This means providing support for long-term adherence to treatment, detecting treatment failure when it arises as soon as possible, and switching people to second-line medication when needed. Data from South Africa suggest that up to 14% of patients fail first-line therapy within five years of starting treatment and need to be switched to second-line therapy [17]; this will vary according to the type of first-line regimen used, background drug resistance, patient adherence and, crucially, the availability of viral load to detect early treatment failure.

Finally, as programmes succeed in identifying people and initiating treatment earlier, and as people living with HIV live longer, health providers will be faced with new challenges of managing HIV infection as a chronic condition. In the US, western Europe and Brazil, as improvements in access to ART have led to important declines in overall mortality and longer life expectancy, healthcare providers have been confronted with an increase in chronic HIV-related conditions, notably neurologic, cardiovascular and chronic hepatic illnesses, and AIDS- and non-AIDS-related malignancies [18–21].

## Ways forward

Improving access to ART and reducing the risk of late presentation to care start with improved uptake of HIV testing. Testing coverage is increasing in most parts of the world but is still suboptimal, particularly among infants, adolescents and key populations. Recognizing the need to improve access to testing, WHO recently recommended an expansion of testing approaches, both within health facilities and in the community [22]; there is also considerable interest in the potential for self-testing to increase access, particularly for populations who may not be normally reached by clinic-based testing approaches [23]. There is some evidence that out-of-clinic testing approaches can also help reach people earlier in their disease progression, thereby reducing the number of patients who present late to care [24]. Nevertheless, a proportion of patients can still be expected to present with advanced disease, and clinical capacity must be retained to provide appropriate diagnosis, treatment and care for this group.

Linkages to ART services for people who test positive have to be strengthened, particularly given the high rate of loss to follow-up between testing and ART initiation [25]. Recent studies have indicated promising approaches for improving linkages from HIV testing to treatment, including point-of-care CD4 testing to speed up eligibility assessment [26], peer support [27] and the offer of community-based testing and treatment [28] to support enrolment and retention in care.

The latest WHO guidelines recommend earlier initiation of ART at CD4 cell counts of ≤500 cells/mm^3^ and immediate ART for certain populations. These recommendations can be expected to reduce overall losses between testing and treatment by reducing the time spent waiting for treatment. Several approaches have been shown to improve adherence and retention for people on ART in a range of settings, including decentralization of services to improve access [29], adherence clubs [30], reduced clinic visits for stable patients [31] and SMS reminders [32]. Ongoing implementation science research is needed to identify simple, inexpensive interventions that work to reduce critical losses to care across a range of settings.

Long-term treatment success depends on effective monitoring to further support adherence and diagnose treatment failure. WHO recommends routine viral load monitoring as the preferred treatment monitoring strategy, though viral load capability remains limited in most resource-limited settings. Efforts are underway to improve access, which can be expected to be further supported through the availability of new point-of-care testing technologies. As viral load monitoring helps both reinforce adherence where this is a challenge and detect treatment failure when it occurs [33], concurrent efforts will be needed to reduce cost and improve access to affordable second- and third-line regimens [34].

The future public health response to HIV in resource-limited settings will have to identify ways to integrate simple and affordable approaches to tackling HIV infection as a chronic condition. Recent research has shown that non-communicable disease programmes can be successfully linked and integrated into HIV programmes [35], and new oral therapies against emerging public health problems, such as hepatitis C virus, provide opportunities for managing this increasingly important co-infection [36]. Such innovations will help inform future WHO guidance in this area.

## Conclusions

Although there are impressive reductions in HIV deaths and new infections globally, this success is not uniform. Focused efforts are needed to support those countries that are struggling to make progress in scaling up ART, and improve access to care for underserved populations. For children, improving access to earlier diagnosis and improved treatment is critical, while for key populations more work is needed to overcome persistent legal and cultural practices that drive stigma and discrimination and limited leadership to prioritize treatment and care.

Strategies to deliver effective HIV care continue to evolve towards greater simplification of treatment. A decade ago, ART consisted of multiple tablets, taken two to three times a day, with common side effects, often administered by doctors and in centralized hospitals. Today, ART, as recommended by WHO and adopted by most high-burden countries, consists of a single pill taken once daily, with treatment administered by nurses in primary care settings, and a progressive evolution towards earlier treatment initiation in line with emerging evidence.

Approaches to ART delivery will continue to evolve as newer drugs, formulations and diagnostics become available, supporting further evolutions towards the management of HIV infection as a chronic condition and sustaining treatment access for increasing numbers of people. In order to maximize the benefit of such innovations and come closer to the goal of zero HIV deaths and, in the longer term, towards an HIV cure, a more nuanced understanding of the evolving nature of both the HIV epidemic and the global response is needed in order to identify those populations that are underserved by the AIDS response. In this way, progress towards zero deaths from HIV cannot be made without progress towards zero HIV discrimination.
